# Prognosis of screen-detected breast cancers: results of a population based study

**DOI:** 10.1186/1471-2407-6-17

**Published:** 2006-01-23

**Authors:** Laura Cortesi, Vincenzo E Chiuri, Silvia Ruscelli, Valeria Bellelli, Rossella Negri, Ivan Rashid, Claudia Cirilli, Antonella Fracca, Ennio Gallo, Massimo Federico

**Affiliations:** 1 Dipartimento di Oncologia ed Ematologia, Università di Modena e Reggio Emilia, Italy; 2 Dipartimento di Diagnostica per Immagini, Università degli Studi di Modena e Reggio Emilia, Italy; 3 Registro Tumori di Modena

## Abstract

**Background:**

The reduced mortality rate from breast carcinoma among women offered screening mammography is demonstrated after 15–20 years of follow-up. However, the assessment of 5-year overall and event-free survival could represent an earlier measure of the efficacy of mammography screening program (MSP).

**Methods:**

All cases of breast cancer diagnosed in the Province of Modena between years 1996 and 2000 in women aged 50 to 69 years, were identified through the Modena Cancer Registry (MCR). Stage of disease and treatment information were obtained from clinical records. All the events occurring up to June 30, 2003 were retrieved by experienced monitors. Five-year overall and event-free survival were the principal end-points of the study.

**Results:**

During a 5-year period, 587 primary breast cancers were detected by the MSP and 471 primary breast cancers were diagnosed out of the MSP. The screen-detected breast cancers were smaller, more likely node negative, with low histological grade, low proliferative activity and positive receptors status. Furthermore, the breast cancer diagnosed through the MSP more frequently received a conservative surgery. The 5-year survival rate was 94% in the screen-detected group, versus 84% in the other group (p = 0.0001). The rate of 5-year event-free survival was 89% and 75% for the MSP participants and not participants, respectively (p = 0.0001).

**Conclusions:**

Our data confirm a favourable outcome of screen-detected breast cancers in terms of five-year overall and event-free survival, which reflect the good quality assurance parameters of the MSP. Finally, a cancer registry should be implemented in every area covered by screening programs.

## Background

Breast cancer is the most common malignancy in women, both in the developed and developing world, with annual incidence rates ranging from 11.8 per 100,000 in eastern China to 86.3 per 100,000 in North America[1]. During recent decades the incidence of breast cancer has increased in Western Europe and the United States, while the mortality decreased[2,3]. Part of the increase in incidence reflects the success of breast cancer screening programs[4]. There is sufficient evidence from randomized trials that inviting women 50–69 years of age to be screened with mammography will eventually reduce their mortality from breast cancer. The first randomized clinical trial performed in New York during the 1960s evaluated the value of "early detection" by physical examination and mammography[5], while the Swedish trials, during the 1970s and 1980s (Malmo, Two-County, Stockholm and Goteborg) used only mammography to find out the independent value of this modality [6-9]. A recent review performed by a committee confirmed their previous results [10]. Even another randomized trial in the United Kingdom (Edinburgh) gave the same results[11]. Mammography screening reduces breast cancer mortality of invited women by 20% to 30% in comparison with controls. Mammography screening makes it possible to detect breast cancer at a subclinical or non-palpable stage. Early detection of such small tumours by screening shows a positive effect on survival because of overall decreased mortality from breast cancer [12-14]. Even after a follow-up period as long as 17 years the expected rate of mortality from breast cancer is reduced[15]. In Modena, Italy, a population-based mammography screening for breast cancer was begun in 1995, providing a biennial screening to women aged 50 to 69 years. However, since the effect on breast cancer mortality will only become evident in the long term, monitoring the early outcomes seemed to us essential in the early phases for early evaluation of the efficacy of the screening program. In fact, several studies have shown that the interval from initial diagnosis to recurrence is a relevant prognostic factor [16,17], since patients with early recurrence tend to do worse than those with late relapse [18].

In the current study we report the results of an exhaustive review of all breast cancers diagnosed between October 1995 and December 2000 in the Province of Modena, in women 50–69 years of age, assessing the rates of small tumors, the patterns of care and the relapse-free, event-free and 5 years survivals in women exposed to screening and in women diagnosed over the same time-interval out of screening.

## Methods

### Data sources

In October, a population-based mammography screening program (MSP) began in the Province of Modena, one of the nine provinces of the Emilia-Romagna region (located in northern Italy). On december 1996, the population of Modena was 613,625, which by the end of 2000 had increased to 632,626, with a density of 235 inhab./sq.km. Out of this population, 80,210 were healthy women, aged 50–69 years. Briefly, all the asymptomatic female residents in the Province of Modena aged 50–69 years were invited to participate in a biannual bilateral two-view screening mammogram, with no direct financial charge to the participant. Women already affected by breast cancer were not invited. From 31^st ^December, 1999 to 31^st ^December, 2000, 48,928 women aged 50–69 years participated in the screening mammography, with an adhesion rate of 61.3%. Up to 31^st ^December, 2000, 43,479 women adhered to the program (adhesion rate of 65.9%).

### Subjects

All the patients with an age ranging from 50 to 69 years diagnosed with breast cancer in the period 1996–2000 were selected from the Modena Cancer Registry (MCR), which has operated since 1988 and collects all new cases of cancers occurring in residents in the Province of Modena. A cross linkage between data from MSP and MCR allowed the identification of cases with invasive or *in situ *breast cancer diagnosed between 1^st ^January, 1996 and 31^st ^December, 2000 who did and did not the MSP. All breast cancer cases were identified as *International Classification of Disease *(9^th ^revision) rubric 174. For every case, data on histology, staging at presentation (based on the 2003 UICC-TNM classification [19] and treatment were obtained from clinical records. All the events (local or distant recurrence, either second breast or non-breast tumours and deaths from breast cancer or from any other cause) that occurred among these women through during the follow-up were retrieved by experienced clinical documentation clerks with active follow-up strategies.

### Statistical analysis

Overall survival was defined as the time from surgery until death from any cause, and cancer-free survival was defined as the time from surgery until the appearance of the first recurrence of cancer, a second cancer, or death from any cause. Survival was estimated by the Kaplan-Meier method, and any differences in survival were evaluated with a stratified log-rank test.

The Statistical Package for Social Sciences (version 10, SPSS^®^) was used for all calculations. Differences were considered to be statistically significant when the P value was 0.05 or less. All statistical tests were two-sided.

The Cox proportional hazards regression model was used in multivariate analysis, to determine whether the identified risk factors independently influenced survival rates. The co-variates selected were: modality of detection (screening vs. non screening), TNM stage (I vs others), chemotherapy (performed or not performed) and hormonal treatment (performed or not performed). Hazard ratios (HR) and 95% exact mid-P confidence intervals (CI) were calculated and the Wald method was used for the significance test.

As shown in table 1, screen-detected breast cancers had a more favorable stage at diagnosis than the non-screen-detected breast cancer. Lead-time bias (i.e., the time interval between cancer detection and death being longer in screen-detected than in non-screen-detected breast cancers, due to early diagnosis) and length bias (i.e., screening being more likely to detect breast cancers with long preclinical phases than rapidly progressive cancers) are two potential sources of bias in the evaluation of the effectiveness of breast cancer screening. In order to limit the effect of lead-time and length bias, information on stage distribution was used as follows:

ts=∑ixi,sdNsd−∑jxj,nsdNnsd
 MathType@MTEF@5@5@+=feaafiart1ev1aaatCvAUfKttLearuWrP9MDH5MBPbIqV92AaeXatLxBI9gBaebbnrfifHhDYfgasaacH8akY=wiFfYdH8Gipec8Eeeu0xXdbba9frFj0=OqFfea0dXdd9vqai=hGuQ8kuc9pgc9s8qqaq=dirpe0xb9q8qiLsFr0=vr0=vr0dc8meaabaqaciaacaGaaeqabaqabeGadaaakeaacqWG0baDdaWgaaWcbaGaem4Camhabeaakiabg2da9maalaaabaWaaabeaeaacqWG4baEdaWgaaWcbaGaemyAaKMaeiilaWIaem4CamNaemizaqgabeaaaeaacqWGPbqAaeqaniabggHiLdaakeaacqWGobGtdaWgaaWcbaGaem4CamNaemizaqgabeaaaaGccqGHsisldaWcaaqaamaaqababaGaemiEaG3aaSbaaSqaaiabdQgaQjabcYcaSiabd6gaUjabdohaZjabdsgaKbqabaaabaGaemOAaOgabeqdcqGHris5aaGcbaGaemOta40aaSbaaSqaaiabd6gaUjabdohaZjabdsgaKbqabaaaaaaa@5089@

and

*c*_*i*,*s *_= *x*_*i*,*s *_- *t*_*s*_

where *t*_**s **_is the mean difference in follow-up time duration between the two groups at stage *s*, for *i *from 1 to *N*_sd _and for *j *from 1 to *N*_**nsd**_, *x*_*i*,**sd **_and *x*_*j*,**nsd **_are durations of follow-up for each woman at each stage in each group and *N*_**sd**_and *N*_**nsd **_are the population size of screen and not screen-detected groups. Then, the correct follow-up time (**c**_**i,s**_) is obtained by subtracting the stage-specific difference from the follow-up time in the screen-detected group.

## Results

### Proportion of cancers detected by the MSP

Between 1^st ^January, 1996 and 31^st ^December, 2000, 587 (55%) new breast cancers were detected among women aged 50–69 attending the MSP. The detection rate was 10%0 (428) in the first, 6.5%0 (152) in the second and 7.9%0 (7) in the third round. The recall rate was higher at the first round (8.2%) than in the second (6.4%) and the third round (4.5%). In the same time frame 471 (45%) breast cancers were diagnosed out of the MSP. Out of these cases, 337 had had a carcinoma before they were first invited by the MSP, 56 arose in women excluded by the screening program since they had a mammography in the last year and 78 arose in women that refused to participate in a screening program. Out of 587 screen-detected breast cancers, which also include 34 interval cases developed in women who had participated in the screening program in the past, 482 (82%) were infiltrating and 105 (18%) were *in situ *carcinomas. Out of the 471 breast cancers non-screen-detected, 429 (91%) were infiltrating and 42 (9%) were in situ carcinomas. The median age at diagnosis was 60 years in both groups. Furthermore the median age at diagnosis in women of 50–69 years in the pre-screening era was 60.5. The age-specific rate of invasive cancers diagnosed each year increased from 304.7 per 100.000 in 1996 to 380.3 per 100.000 in 2000. The age-specific rate of in situ carcinoma diagnosed each year increased from 31.9 per 100.000 in 1996 to 49.7 per 100.000 in 2000. As already reported [20] the incidence rates increased only in the age group 50–69 years.

### Prognostic factors

The clinical pathological factors for the two cohorts are shown in Tab. 2. There were significant differences between the screen-detected and non-screen-detected cancers with respect to clinical/pathological prognostic factors (all *P *< 0.0001). The screen-detected tumours were smaller sized, were more likely to be grading I-II, pathologically confirmed node negative disease and low proliferative activity. There was also a significant difference in the percentage in women with positive hormonal status (84% and 73%, respectively; P = 0.02).

Concerning the characteristics of interval cancers, they had a mean size of 18 mm, the nodal status and the hormonal receptors were positive in 24% and 70% of cases, respectively, and the proliferative activity was high in 34% of tumours. Finally the tumours was poorly differentiated (GIII) in 45% of cases.

### Survival data

The median follow-up was 52 months in both groups, with a 25% of patients followed for more than 66 months. Survival data were evaluated only for invasive cancers, i.e. for 482 patients exposed to screening and 429 in the non-screen exposed group. Six (1%) and thirty-nine (9%) patients were lost at the follow-up in the study and control group, respectively. At time of last follow-up 22 women had died in the screen-detected (20 for breast cancer and 2 for other causes) and 60 in the non-screen-detected group (19 for breast cancer recurrences and 41 for others causes). The overall survival rates in the two groups differed significantly on the basis of the stratified log-rank test. As expected, the majority of deaths occurred in patients with breast cancer non- screen-detected (p < 0.0001).

**Figure 1:**
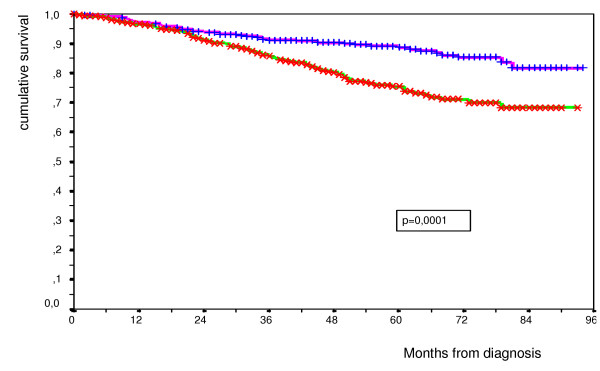
*5-year event-free survival*. 5-year event-free survival values between MSP participants and non-participants were equal to 89% and 75% respectively (Log Rank = 19.99). [ = MSP participants;  = MSP non-participants]

**Figure 2:**
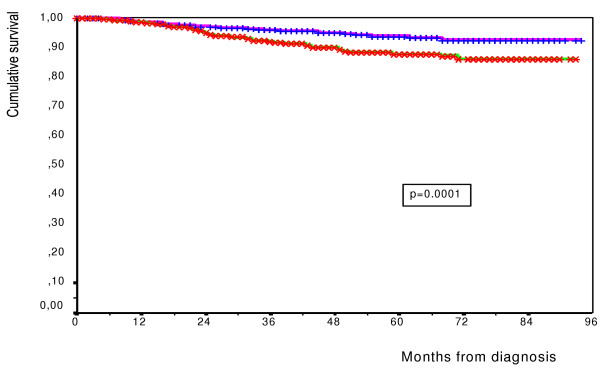
*5-yearOS*. 5-year OS between MSP participants and non-participants was equal to 94% and 84% respectively (Log Rank = 14.99). [ = MSP participants  = MSP non-participants]

At the time of the present analysis a total of 131 events were registered, including 75 recurrences, 10 second breast cancers, 25 different second malignancies, and 21 deaths not related to breast cancer or its treatment. We observed 48/482 (9.6%) events in the screen-detected group and 83/429 (22.6%) in the non-screen-detected group, with a statistically significant difference (p < 0.0001). A significant difference was also observed in the number of disease recurrences (33 in the screen-detected group and 42 in the non-screen-detected group, p = 0.02) and of second breast cancers (1 vs 9, p = 0.02) (Tab. 3). As a result of these differences, the 5-year event-free survival was equal to 89% and 75% among the MSP participants and non-participants, respectively (p = 0.0001) (Fig. 1). The 5-year overall survival was 94% in the screen-detected group and 84% in the non screen-detected group and this difference was statistically significant (p = 0.0001) also after adjustment for calculated lead-time and length biases (Fig. 2). In both analyses the interval cancers were also included. In univariate analysis of overall survival, factors associated with a good prognosis were: detection by screening (p < 0.0001), TNM stage I (p < 0.0001), chemotherapy (p < 0.0001) and hormonal therapy (p = 0.25). In a multivariate analysis diagnosis through the MSP, TNM stage I and hormonal treatment remained an independent prognostic value (Tab. 4).

**Figure 3:**
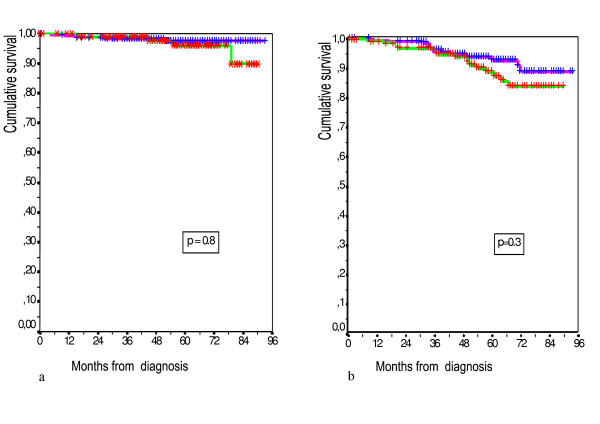
*5-year survival of pT1a+b (3a) and pT1c (3b)*. 5-year OS values of pT1a+b (a) and pT1c (b) breast cancers between MSP participants and non-participants were equal to 98% and 96% (Log Rank = 0.76) respectively (a) and 93% and 89% (Log Rank = 1.57) respectively (b). [ = MSP participants;  = MSP non-participants]

The 5-year estimated survival rates were 98% and 96% = 0.8 (Fig. 3a) for T1a-b stage, respectively in the screen-detected and non-screen-detected group. Also for T1c stage, the 5-year estimated survival rates were similar in the two group (93% vs. 89%, respectively = 0.3). (Fig. 3b). No significant difference in the 5-year survival rates of the two stages was found between the two groups. This finding shows that the prognosis of small tumours is not influenced by the modality of diagnosis.

**Figure 4:**
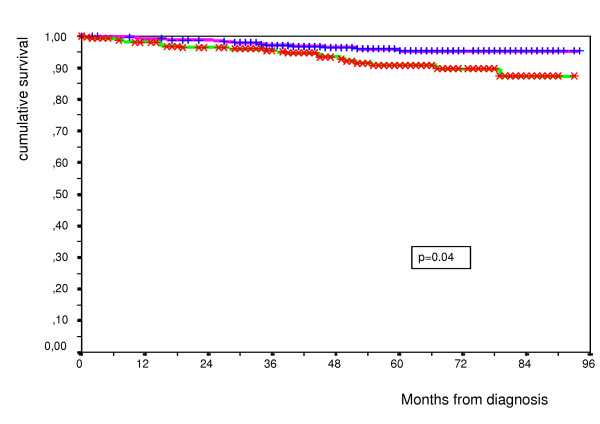
*5-year survival in N- BC*. 5-year OS values of N- breast cancers between MSP participants and non-participants were equal to 95% and 91% respectively (Log Rank = 5.74). [ = MSP participants;  = MSP non-participants]

**Figure 5:**
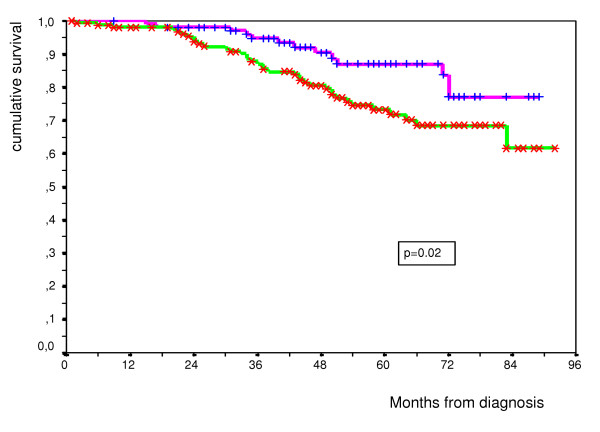
*5-year survival in N+ BC*. 5-year OS values of N+ breast cancers between MSP participants and non-participants were equal to 87% and 73% respectively (Log Rank = 5.02). [ = MSP participants;  = MSP non-participants]

However, considering node-negative cases as a whole, the 5-year survival rates were 95% in the screen-detected group and 91% in the non-participants to the MSP (Fig. 4). The difference between the two groups was statistically significant (p = 0.04) depending on the higher rate of smaller tumors in the MSP group. Also the 5-year survival rates for the node-positive breast cancers showed a significant difference between the two groups (87% in the screen-detected breast cancer and 73% in the non-screen-detected (p = 0.02)(Fig. 5).

## Discussion

The present study has shown that breast cancers detected through MSP are usually diagnosed at an early stage and are associated with a very favourable behaviour of the disease and an excellent prognosis, in terms of 5-year event-free survival and Overall Survival. Moreover, the study has also suggested that a population-based cancer registry is a relevant tool in the evaluation of a cancer screening program.

The decision to offer regular mammographic screening is based on the demonstration of reduced breast cancer-specific mortality in prospective randomized clinical trials [21-24]. In the randomized trials of breast screening, comparison of the prognostic profile of cancers detected in the invited as compared with the control groups has identified surrogate intermediate endpoints associated with the mortality reductions in those trials. These endpoints include such factors as the proportion of cancers smaller than 15 mm diameter or absence of lymph node involvement at diagnosis. It has been suggested that these intermediate endpoints might be useful to predict the likelihood that an organized breast screening program will achieve its mandate of mortality reduction on a population basis [25]. The prognostic profile of screen-detected breast cancer within the MSP not only meets the targets derived from the randomized trials, but also demonstrates a significant advantage in terms of initial therapy and event-free survival when compared to other breast cancers diagnosed in the same geographically-defined population over the same time period.

In fact, since the beginning of the organized mammography screening program, an increasing proportion of women with newly diagnosed breast cancer each year (76 in 1996, 100 in 1997, 131 in 1998, 173 in 1999, 175 in 2000) have been MSP participants. The cancers diagnosed in women within four years of the screening program were histologically more favourable (GI-II, hormonal receptors positive, low proliferative activity), smaller (13 mm vs 19 mm) and less likely to have spread to axillary lymph nodes compared to breast cancer diagnosed out of the screening over the same time frame.

Since the crude breast cancer-specific mortality rate cannot be used for an immediate evaluation of the impact of screening mammography, we have used Event-Free Survival at 5 years as a measure of success of the MSP. We considered as events local or distant recurrence of breast cancer, second breast cancer, second cancer other than breast, and death from any cause. The rate of recurrences and second breast cancers, but not of other second malignancies or deaths, was significantly lower in the screen-detected group. As a consequence we observed a statistically significant and clinically meaningful 14% improvement in the 5-year event-free survival of women diagnosed by the MSP compared with those diagnosed out of the screening (89% vs. 75%). A difference was also observed in the subset of node negative tumors, where the 5-year survival rates are 95% in the screen-detected group and 91% in the non participants to the MSP. These results correlate with data from a recent study performed by Joensuu, in which a 5-year survival rate of 97% was observed in women with node negative tumors [26]. In addition Joensuu and colleagues found a significant correlation between tumor size and prognosis. In fact, the 20-year survival rate was 93% for tumors of one cm or less, and only 75% for tumors of more than one cm in size. On the basis of that study we can predict that the excellent 5-year survival rate of 94% observed for tumors detected by the MSP (48% of which have a maximum diameter of one centimeter or less) will translate in a long term (i.e. 10 years) survival exceeding 90%.

Welch et al. [27] and Baum [28], state that 5-years survival rate is not sufficient to demonstrate the effectiveness of a screening program This period is considered too brief to by-pass the lead-time bias, that is the earlier detection of a breast cancer, which increases the survival from the time diagnosis to the time of death, without influencing the natural history of the disease. Calculating the 5-year survival rates after adjustment for lead-time and length bias confirmed our finding of a better prognosis of screen-detected cancers. The analysis of mean follow-up duration for every stage showed a superimposable period between the two groups, particularly for stage I BC, which were the most frequently detected cancers in the screen-detected group. Although the MSP seem sufficiently cost-effective, we have to realize that 29% of the tumours were not detected by screening. In fact, the MSP not only missed a small number of interval cancers (n = 34) but also did not cover the cancers occurring in patients excluded for screening because of a recent mammogram outside the MSP (n = 56) and in women who refused to participate in the screening program. Our results confirm that the characteristics of interval cancers are intermediate, between screen and non-screen detected tumors, in terms of size (17.7 mm), nodal status (24% positive), hormonal receptors (70% positive) and high proliferative activity (34%). Overall the sensitivity of our screening program, considered as a proportion of screen-detected cancers in all cancers diagnosed in the target population was 55%. However, the performance of the MSP compares favourably with the recommendations of the European Commission for quality assurance in mammography screening [29], which indicate a good detection rate at the first round when it is more than 3.5 fold of the incidence rate before the screening (2.6%0) and more than 1.5 fold at the subsequent rounds. Finally, we exclude that a class bias could have influenced our results since the number of women belonging to a low social class in the province of Modena according to the Statistic Service is very low (0.83%). Even though there is no direct information on social class status of population invited to the MSP, in an interview performed for a cervical screening program in Modena province, no differences between women belonging to a high or low social class in terms of participation to the study were found. In fact almost all women participated in the study, whereas in many other countries only women from the higher socioeconomic and educational strata are more likely to accept the invitation to screen and to participate in other health-promoting patterns of behaviour [30].

Our data clearly show that the major cause for the decreasing breast cancer mortality is the smaller tumour size, associated with the earlier detection by the MSP. Moreover, the chemotherapy was not retained as an independent prognostic factor, confirming that the magnitude of impact of chemotherapy is very likely less relevant compared to the effect of early detection. Despite that, in our series of screen-detected breast cancers, a very high rate of patients were treated with chemotherapy, even if the most used regimen was CMF.

Finally, we would remark that our study was made possible and facilitated only by the existence of a population-based cancer registry in the area. Through the registry we recognized all new cases of cancers in women aged 50–69 years and achieved information on follow-up status. We consider a cancer registry an important tool for an adequate monitoring of the screening programs.

## Conclusion

In conclusion, our data show the effectiveness of the MSP in terms of early diagnosis, more favorable behaviour of the tumor and better prognosis, even if only 65% of the age-eligible female were partecipating in this program in 1996–2000. This analysis has pointed out the need and potential value of substantially increasing recruitment to the MSP to 70% or more, as achieved in other geographic areas.

## Competing interests

The author(s) declare that they have no competing interests.

## Authors' contributions

LC participated in the design of the study and drafted the manuscript.

VEC acquired the data and performed the statistical analysis.

SR participated in the design of the study.

VB performed the mammographic screening.

RC acquired the mammographic screening data.

IR acquired the data of the Modena Cancer Registry.

CC participated in the statistical analysis.

AF developed the method of eliminating the lead-time and length biases.

EG coordinated the Screening Mammography Program in Modena.

MF designed the study and revised the final manuscript.

All authors read and approved the final manuscript.

## Pre-publication history

The pre-publication history for this paper can be accessed here:



## Figures and Tables

**Table 1:** Mean follow-up between the two groups and relative differences between patients at every stage

Stage	Distribution of those exposed to screening	Time of mean follow-up in thoxe exposed to screening (months)	Distribution in non screen-detected	Time of mean follow-up in non-screen-detected (months)	Differences between mean follow-up (months)
I	357	54.13	199	54.01	0.12
II	115	51.39	156	48.49	2.9
III	2	50.58	30	49	1.56
IV	8	32.57	26	36.32	-3.75

**Table 2:** Prognostic factors' distribution among women exposed to screening and non-screen-detected invasive breast cancers

Prognostic Factor	Exposed to screening n(%)	Non-screen-detected n(%)	p-value
Tumour size (mm.)			
1–10	231 (48)	102 (24)	
11–20	218 (45)	167 (39)	
21–50	33 (7)	140 (32)	
> 50	--	20 (5)	
			
Mean Size	13	19	< 0.0001
Positive Nodal Status	93 (19)	165 (38)	< 0.0001
Positive Hormonal Status	403 (84)	313 (73)	< 0.05 (0.02)
Proliferative Activity			< 0.0001
LOW	343 (74)	236 (61)	
HIGH	118 (26)	150 (39)	
Grading			<0.0001
1	108 (25)	76 (21)	
2	209 (49)	143 (40)	
3	113 (26)	142 (39)	

**Table 3:** Outcome and type of events that occurred in women, aged 50–69 years, with breast cancer diagnosed between 1996 and 2000 in the Province of Modena by screening status

	Screen-detected n(%)	Non screen-detected n(%)	p-value
EVOLUTION			< 0.0001
Alive	454 (94)	330 (77)	
Dead	22 (5)	60 (14)	
Lost at follow-up	6 (1)	39 (9)	
RECURRENCE	33 (6.8)	42 (9.7)	0.02
Local	8 (1.6)	12 (2.8)	
Distant	25 (5.1)	30 (6.9)	
SECOND BREAST CANCER	1 (0.2)	9 (2)	0.02
BREAST CANCER SPECIFIC DEATH	20 (4)	41(9)	<0.05
OTHER SECOND MALIGNANCIES	15 (3)	16 (4)	NS
DEATH FROM OTHER CAUSES	2 (0.4)	19 (4.4)	<0.0001
TOTAL EVENTS	48(9.9)	83(19)	<0.0001

**Table 4:** Factors with independent prognostic value of survival

Factor	HR	95%CI	P value
DIAGNOSIS INSIDE MSP			
No	1(referent)		
Yes	0.58	0.36–0.92	0.023
STAGE			
II,III, IV	1(referent)		
I	0.16	0.08–0.30	0.0001
HORMONAL THERAPY			
No	1(referent)		
Yes	0.50	0.32–0.78	0.002
